# Impact of functional disability on health-care use and medical costs among cancer survivors

**DOI:** 10.1093/jncics/pkad059

**Published:** 2023-08-16

**Authors:** Hyo Jung Tak, Ronnie D Horner, Min Sok Lee, Ya-Chen Tina Shih

**Affiliations:** Department of Health Services Research and Administration, University of Nebraska Medical Center, Omaha, NE, USA; Department of Health Services Research and Administration, University of Nebraska Medical Center, Omaha, NE, USA; Department of Economics, University of Chicago, Chicago, IL, USA; Jonsson Comprehensive Cancer Center and Department of Radiation Oncology, David Geffen School of Medicine, University of California Los Angeles, Los Angeles, CA, USA

## Abstract

**Background:**

Cancer survivors with a disability are among the most vulnerable in health status and financial hardship, but no prior research has systematically examined how disability modifies health-care use and costs. This study examined the association between functional disability among cancer survivors and their health-care utilization and medical costs.

**Methods:**

We generated nationally representative estimates using the 2015-2019 Medical Expenditure Panel Survey. Outcomes included use of 6 service types (inpatient, outpatient, office-based physician, office-based nonphysician, emergency department, and prescription) and medical costs of aggregate services and by each of 6 service types. The primary independent variable was a categorical variable for the total number of functional disabilities. We employed multivariable generalized linear models and 2-part models, adjusting for sociodemographics and health conditions and accounting for survey design.

**Results:**

Among cancer survivors (n = 9359; weighted n = 21 046 285), 38.8% reported at least 1 disability. Compared with individuals without a disability, cancer survivors with 4 or more disabilities experienced longer hospital stays (adjusted average marginal effect = 1.14 days, 95% confidence interval [CI] = 0.55 to 1.73), more visits to an office-based physician (average marginal effect = 1.43 visits, 95% CI = 0.51 to 2.35), and a greater number of prescriptions (average marginal effect = 12.1 prescriptions, 95% CI = 9.27 to 15.0). Their total (average marginal effect = $9537, 95% CI = $5713 to $13 361) and out-of-pocket (average marginal effect = $639, 95% CI = $79 to $1199) medical costs for aggregate services were statistically significantly higher. By type, disability in independent living was most strongly associated with greater costs for aggregate services.

**Conclusions:**

Cancer survivors with a disability experienced greater health-care use and higher costs. Cancer survivorship planning for health care and financial stability should consider the patients’ disability profile.

##  

Cancer survivors with a disability make up one of the most vulnerable cohorts in terms of their health status and financial hardships. Cancer survivors often encounter physical, emotional, and financial tribulations (1-3). These conditions could be aggravated by preexisting or newly triggered disability because disability is associated with higher risk of poor health outcomes (4-6) and greater need for health services to treat them (7).

Empirical evidence on the association of disability with health services utilization and medical costs is scarce in the cancer care literature (8,9). Prior research has primarily focused on the association of preexisting disability with disparities in cancer screening (10,11) and treatment (12-14) and on productivity loss resulting from postcancer disability (15,16). Although numerous studies have investigated unmet health-care needs and cancer-related financial hardship, these studies compared individuals with and without cancer regardless of disability conditions (2,8,17,18). Consequently, the current literature offers little information about whether cancer survivors who have disabling conditions experience greater use of health-care and greater financial hardship through higher medical costs.

Clearly, prevention and management of disability—one of the overarching goals of Healthy People 2020 (19,20)—is particularly important for cancer survivors. One of the methodological difficulties for research into this topic, however, is the lack of a standard case definition of *disability;* consequently, both type and estimated prevalence of disability have varied widely in this population (21-24). To address this concern, the Patient Protection and Affordable Care Act adopted the World Health Organization’s guidelines for standard disability identification (25), and the US Department of Health and Human Services (HHS) codified survey questions to uniformly define 6 functional disability conditions (26). These steps allowed us to identify cancer survivors with disabilities using a standardized definition, a crucial step toward understanding how disability affects health-care experience and health status in these individuals.

Using nationally representative data, our goals were to determine whether the prevalence of functional disability per the HHS definition was higher among those with than without a cancer history. Then, we assessed the association of functional disability with health services utilization, total medical costs, and out-of-pocket medical costs among cancer survivors. Findings from this research advance our understanding of the health-care needs and financial burden among those who have both a history of cancer and a disability.

## Methods

### Data and study population

Our data source was the 2015-2019 Medical Expenditure Panel Survey Household Component Full Year Consolidated Public Use File (MEPS-FC) (27). MEPS-FC is a large-scale, nationally representative survey of noninstitutionalized civilians in 50 states and Washington, DC. For each respondent, MEPS-FC collected data on individual and family characteristics, health insurance, health conditions, health services utilization, and costs.

In MEPS-FC, cancer survivor status was determined by the respondent answering yes to the question “Have you ever been told by a doctor or other health professionals that you had cancer or a malignancy of any kind?” where the diagnosed cancer was not solely a nonmelanoma skin cancer (28).

To compare disability status between individuals with and without a cancer history, the study population was restricted to adults (aged ≥18 years). MEPS-FC collected cancer-related information for adults only. Then, to investigate the association of disability with health services utilization and medical costs among cancer survivors, the study population was restricted to adults with a history of cancer (Supplementary Figure, available online).

This study was exempt from full review by the institutional review board at the University of Nebraska Medical Center.

### Outcomes

Three sets of outcomes were assessed: annual health services utilization, total medical costs, and out-of-pocket medical costs.

We quantified annual health services utilization in terms of 6 service categories: hospital length of stay (LOS; inpatient care), number of visits to an outpatient department, office-based physician, office-based nonphysician, and emergency department (ED), and number of prescriptions.

Annual total medical costs were calculated as the sum of costs paid by insurance and out-of-pocket costs. Out-of-pocket medical costs included deductibles and coinsurance/copayment. Total and out-of-pocket costs were measured for each of the 6 services and the aggregated costs, which included the 6 service categories plus home health care, dental care, and other medical equipment and services. All costs were adjusted to 2019 US dollars using the Personal Medical Care Services Price Index (29).

### Primary independent variables

The primary independent variable was a categorical variable indicating total number of functional disabilities reported by each individual (0, 1, 2, 3, ≥4). Alternatively, we used 6 binary variables for each condition (hearing, vision, cognition, ambulation, self-care, independent living) to examine potentially differential impact by type of disability. The survey questions codified by HHS (26) and employed by MEPS-FC are presented in the Supplementary Table (available online).

### Other explanatory variables

Other variables in the multivariable regression analyses included age categories (18-40, 41-50, 51-60, 61-70, 71-80, 81-85 years), sex, race/ethnicity (Hispanic, non-Hispanic Black, non-Hispanic Other, non-Hispanic White), marital status, education (less than high school, high school, any college, any graduate), employment status, family income, and insurance status (private, Medicare only, Medicare and private supplemental, Medicare and Medicaid, Medicaid or other public, no insurance). We also adjusted for general health status (excellent or very good, good, fair or poor), the 5 chronic conditions most prevalent among our final study population (high blood pressure, high cholesterol, arthritis, heart disease, diabetes), region (Northeast, Midwest, South, West), and calendar year.

### Statistical analyses

Among all adults, we used Pearson χ^2^ tests to compare characteristics of individuals with vs without a history of cancer.

Among cancer survivors, we used *t* tests and Pearson χ^2^ tests to assess whether there was a systematic difference in each continuous and categorical variable, respectively, by disability status.

For multivariable regression analyses, we employed a negative binomial model for health services utilization (counts of visits to office-based physicians and nonphysicians, number of prescriptions) and a generalized linear model with a log-link function and γ distribution (30) for total medical costs (aggregate services, office-based physicians and nonphysicians, prescription drugs). For other service types (inpatient, outpatient department, ED), we used a 2-part model (31,32) because these outcomes included at least 50% of zeros. In the first part of the 2-part model, a logistic model was used to estimate none vs any usage/costs. In the second part, a negative binomial model and a generalized linear model with a log-link function and γ distribution were employed for health services utilization and total costs, respectively. For out-of-pocket costs, we applied a generalized linear model with a log-link function and γ distribution for aggregate services and prescriptions and a 2-part model for other service types.

All regression analyses were conducted for 2 model specifications: model 1 used a categorized variable of the total number of disability, whereas model 2 included 6 binary variables for each disability.

We conducted a sensitivity analysis in which we split the age category 18-40 years into ages 18-30 and 31-40 years, then repeated the analysis.

The estimated coefficients were converted into average marginal effects for ease of interpretation (33). Average marginal effects represent the differences in the adjusted predicted outcomes between the comparison and the reference group (eg, difference in the adjusted predicted number of prescriptions between individuals with and without a disability). Average marginal effect is considered statistically significant if its 95% confidence interval (CI) does not include zero.

All statistical analyses accounted for complex survey design in MEPS-FC (probability weight, primary sampling unit, strata) to generate nationally representative estimates. All statistical tests were 2-sided, and *P* ≤ .05 was considered statistically significant. Analyses were performed using Stata MP, version 16.1, statistical software (StataCorp LLC, College Station, TX).

## Results

### Individual characteristics by cancer history among adults

The total sample size was 115 039, representing 245 366 271 US adults (Table 1). Among them, 9359 (8.6%, weighted percentage) had a history of cancer. Compared with individuals without a history of cancer, a statistically significantly greater proportion of cancer survivors had any functional disability (16.1% vs 38.8%) and any specific type of disability (*P *<* *.001 for all).

**Table 1.** Functional disability, demographics, socioeconomic status, general health status, and chronic conditions among all adults and by history of cancer^a^

	Overall	History of cancer		
		No	Yes	
	% (95% CI)	% (95% CI)	% (95% CI)	
	(N = 115 039)	(n = 105 680)	(n = 9359)	*P*
Total No. of disabilities				<.001
0	82.0 (81.5 to 82.5)	83.9 (83.4 to 84.4)	61.2 (59.6 to 62.8)	—
1	10.1 (9.7 to 10.4)	9.2 (8.9 to 9.5)	19.2 (18.1 to 20.4)	—
2	4.0 (3.8 to 4.1)	3.5 (3.3 to 3.6)	9.4 (8.6 to 10.1)	—
3	2.2 (2.0 to 2.3)	1.9 (1.7 to 2.0)	5.3 (4.8 to 5.8)	—
≥4	1.8 (1.7 to 1.9)	1.5 (1.4 to 1.6)	4.9 (4.3 to 5.5)	—
Disability type				
Hearing	5.4 (5.1 to 5.6)	4.6 (4.3 to 4.8)	13.8 (12.8 to 14.9)	<.001
Vision	2.8 (2.6 to 3.0)	2.5 (2.3 to 2.7)	6.1 (5.5 to 6.8)	<.001
Cognition	6.2 (5.9 to 6.5)	5.7 (5.5 to 6.0)	11.5 (10.6 to 12.4)	<.001
Ambulation	10.0 (9.7 to 10.4)	8.7 (8.3 to 9.0)	24.5 (23.3 to 25.8)	<.001
Self-care	2.7 (2.5 to 2.8)	2.3 (2.2 to 2.4)	6.7 (6.0 to 7.4)	<.001
Independent living	5.3 (5.1 to 5.5)	4.6 (4.4 to 4.9)	12.7 (11.7 to 13.6)	<.001
Age category, y				<.001
18-40	39.5 (38.8 to 40.2)	42.6 (41.9 to 43.3)	7.1 (6.3 to 7.9)	—
41-50	16.1 (15.6 to 16.5)	16.7 (16.3 to 17.2)	9.0 (8.0 to 10.0)	—
51-60	17.3 (16.9 to 17.8)	17.2 (16.8 to 17.7)	18.3 (17.0 to 19.7)	—
61-70	14.5 (14.1 to 15.0)	13.4 (13.0 to 13.8)	26.3 (24.9 to 27.8)	—
71-80	8.3 (8.0 to 8.7)	6.8 (6.5 to 7.1)	24.4 (23.1 to 25.7)	—
81-85	4.2 (4.0 to 4.5)	3.2 (3.0 to 3.4)	14.8 (13.6 to 16.0)	—
Women	51.8 (51.5 to 52.2)	51.2 (50.8 to 51.6)	58.7 (57.2 to 60.2)	<.001
Race and ethnicity				<.001
Hispanic	16.0 (14.9 to 17.1)	16.8 (15.6 to 17.9)	7.4 (6.7 to 8.2)	—
Non-Hispanic Black	11.8 (11.0 to 12.6)	12.2 (11.4 to 13.0)	7.6 (6.7 to 8.5)	—
Non-Hispanic other	9.0 (8.3 to 9.7)	9.4 (8.7 to 10.1)	4.5 (3.8 to 5.2)	—
Non-Hispanic White	63.3 (61.9 to 64.6)	61.6 (60.3 to 63.0)	80.5 (79.2 to 81.9)	—
Married	52.3 (51.6 to 53.0)	51.8 (51.1 to 52.5)	57.1 (55.3 to 59.0)	<.001
Education				<.001
Less than high school	12.9 (12.4 to 13.5)	13.0 (12.5 to 13.6)	12.0 (10.9 to 13.0)	—
High school	28.4 (27.8 to 29.1)	28.4 (27.7 to 29.1)	28.8 (27.4 to 30.3)	—
Any college	46.1 (45.4 to 46.8)	46.2 (45.5 to 46.9)	44.6 (42.9 to 46.4)	—
Any graduate	12.6 (12.0 to 13.2)	12.4 (11.8 to 13.0)	14.6 (13.3 to 15.8)	—
Employed	70.1 (69.4 to 70.8)	72.7 (72.1 to 73.4)	41.9 (40.1 to 43.7)	<.001
Family income				<.001
Bottom quartile	20.2 (19.7 to 20.7)	20.6 (20.1 to 21.1)	15.2 (14.1 to 16.3)	—
Second quartile	22.0 (21.5 to 22.5)	21.4 (20.9 to 21.9)	28.2 (26.8 to 29.6)	—
Third quartile	26.1 (25.7 to 26.6)	26.2 (25.7 to 26.7)	25.8 (24.6 to 27.0)	—
Top quartile	31.7 (30.9 to 32.5)	31.8 (31.0 to 32.6)	30.7 (29.2 to 32.3)	—
Insurance status				<.001
Private	54.9 (54.0 to 55.8)	57.3 (56.3 to 58.2)	30.0 (28.2 to 31.7)	—
Medicare only	10.8 (10.3 to 11.2)	9.1 (8.7 to 9.5)	28.7 (27.2 to 30.3)	—
Medicare and private	9.2 (8.9 to 9.6)	7.8 (7.4 to 8.1)	25.0 (23.4 to 26.5)	—
Medicare and Medicaid	2.9 (2.7 to 3.1)	2.6 (2.4 to 2.8)	6.5 (5.7 to 7.3)	—
Medicaid and other public	10.3 (9.8 to 10.9)	10.7 (10.1 to 11.3)	6.2 (5.5 to 6.9)	—
No insurance	11.8 (11.3 to 12.3)	12.6 (12.0 to 13.1)	3.7 (3.2 to 4.2)	—
Health status				<.001
Excellent, very good	59.0 (58.4 to 59.7)	60.4 (59.7 to 61.1)	44.0 (42.4 to 45.6)	—
Good	28.5 (28.0 to 29.0)	28.1 (27.6 to 28.6)	32.5 (31.1 to 33.8)	—
Fair, poor	12.5 (12.1 to 12.9)	11.5 (11.1 to 11.9)	23.5 (22.3 to 24.8)	—
High blood pressure	32.5 (31.9 to 33.1)	30.2 (29.6 to 30.8)	56.9 (55.3 to 58.5)	<.001
High cholesterol	30.1 (29.6 to 30.6)	27.9 (27.4 to 28.4)	53.6 (52.0 to 55.2)	<.001
Arthritis	25.5 (24.9 to 26.1)	23.0 (22.4 to 23.6)	52.6 (50.8 to 54.3)	<.001
Heart disease	14.2 (13.8 to 14.6)	12.7 (12.3 to 13.0)	31.1 (29.5 to 32.8)	<.001
Diabetes	10.3 (10.0 to 10.6)	9.5 (9.2 to 9.9)	18.6 (17.3 to 19.9)	<.001
Region				.09
Northeast	17.7 (16.3 to 19.1)	17.7 (16.2 to 19.1)	18.1 (16.1 to 20.2)	—
Midwest	20.9 (19.7 to 22.1)	20.8 (19.5 to 22.0)	22.3 (20.5 to 24.2)	—
South	37.7 (36.0 to 39.4)	37.7 (36.0 to 39.5)	37.4 (34.9 to 39.9)	—
West	23.7 (22.3 to 25.1)	23.9 (22.4 to 25.3)	22.1 (20.2 to 24.1)	—
Year				.36
2015	19.8 (19.0 to 20.6)	19.8 (18.9 to 20.6)	20.3 (19.0 to 21.7)	—
2016	20.0 (19.2 to 20.7)	19.9 (19.1 to 20.7)	20.3 (19.2 to 21.4)	—
2017	20.0 (19.6 to 20.4)	20.1 (19.7 to 20.5)	19.1 (18.3 to 20.0)	—
2018	20.1 (19.1 to 21.1)	20.1 (19.1 to 21.1)	20.1 (18.8 to 21.3)	—
2019	20.1 (19.4 to 20.9)	20.1 (19.4 to 20.9)	20.2 (19.0 to 21.4)	—

a Weighted total N = 245 366 271. Percentages and 95% CIs were adjusted for survey design (probability weight, primary sampling unit, strata). CI = confidence interval.

### Health services utilization and medical costs by disability among cancer survivors

Among 9359 cancer survivors (weighted n = 21 046 285), ambulatory disability (24.5%) was the most prevalent type of functional disability, followed by hearing (13.8%), independent living (12.7%), and cognition (11.5%) (Table 1).

Compared with cancer survivors without a disability, a statistically significantly greater proportion of those with any disability were older and covered by Medicare, had chronic diseases, and reported that their health was fair or poor. Moreover, proportions of those who were married, were employed, and had higher educational attainment and family income were statistically significantly lower among cancer survivors with disability (*P *<* *.001 for all) (Table 2).

**Table 2.** Functional disability, demographics, socioeconomic status, general health status, and chronic conditions by disability status among cancer survivors (n = 9359)^a^

	Disability status		
	No, % (95% CI)	Yes, % (95% CI)	
	(n = 5513)	(n = 3846)	*P*
Total No. of disabilities			—
0	100 (100 to 100)	0.0 (0.0 to 0.0)	—
1	0.0 (0.0 to 0.0)	49.6 (47.4 to 51.8)	—
2	0.0 (0.0 to 0.0)	24.1 (22.4 to 25.9)	—
3	0.0 (0.0 to 0.0)	13.7 (12.4 to 15.0)	—
≥4	0.0 (0.0 to 0.0)	12.6 (11.2 to 14.0)	—
Disability type			—
Hearing	0.0 (0.0 to 0.0)	35.7 (33.6 to 37.8)	—
Vision	0.0 (0.0 to 0.0)	15.8 (14.2 to 17.4)	—
Cognition	0.0 (0.0 to 0.0)	29.6 (27.7 to 31.6)	—
Ambulation	0.0 (0.0 to 0.0)	63.3 (61.1 to 65.5)	—
Self-care	0.0 (0.0 to 0.0)	17.2 (15.6 to 18.9)	—
Independent living	0.0 (0.0 to 0.0)	32.7 (30.5 to 34.8)	—
Age category, y			<.001
18-40	9.0 (7.9 to 10.1)	4.1 (3.0 to 5.2)	—
41-50	11.5 (10.1 to 12.9)	4.9 (4.0 to 5.9)	—
51-60	21.7 (19.9 to 23.5)	13.1 (11.5 to 14.6)	—
61-70	28.6 (26.7 to 30.4)	22.8 (20.8 to 24.7)	—
71-80	21.8 (20.2 to 23.4)	28.6 (26.6 to 30.6)	—
81-85	7.5 (6.5 to 8.4)	26.5 (24.2 to 28.7)	—
Women	59.5 (57.5 to 61.5)	57.4 (55.3 to 59.5)	.136
Race and ethnicity			.134
Hispanic	8.0 (7.0 to 8.9)	6.6 (5.6 to 7.6)	—
Non-Hispanic Black	7.3 (6.3 to 8.3)	8.0 (6.8 to 9.3)	—
Non-Hispanic other	4.3 (3.6 to 5.1)	4.7 (3.6 to 5.8)	—
Non-Hispanic White	80.4 (78.8 to 82.0)	80.7 (79.0 to 82.4)	—
Married	62.7 (60.7 to 64.8)	48.3 (45.7 to 50.9)	<.001
Education			<.001
Less than high school	8.0 (7.0 to 9.0)	18.2 (16.5 to 19.9)	—
High school	27.3 (25.5 to 29.0)	31.3 (29.1 to 33.5)	—
Any college	47.7 (45.7 to 49.7)	39.7 (37.4 to 42.1)	—
Any graduate	17.0 (15.4 to 18.5)	10.8 (9.2 to 12.3)	—
Employed	56.4 (54.2 to 58.5)	19.0 (17.1 to 20.8)	<.001
Family income			<.001
Bottom quartile	15.3 (13.9 to 16.6)	26.3 (24.3 to 28.2)	—
Second quartile	18.6 (17.1 to 20.0)	32.1 (30.1 to 34.1)	—
Third quartile	28.7 (27.2 to 30.3)	24.1 (22.3 to 26.0)	—
Top quartile	37.5 (35.5 to 39.5)	17.5 (15.8 to 19.2)	—
Insurance status			<.001
Private	41.7 (39.4 to 44.0)	11.5 (9.8 to 13.1)	—
Medicare only	22.9 (21.2 to 24.6)	37.9 (35.6 to 40.3)	—
Medicare and private	22.6 (20.8 to 24.3)	28.8 (26.6 to 30.9)	—
Medicare and Medicaid	2.5 (2.0 to 3.0)	12.7 (11.1 to 14.4)	—
Medicaid and other public	5.9 (5.0 to 6.9)	6.6 (5.5 to 7.6)	—
No insurance	4.4 (3.6 to 5.2)	2.5 (1.9 to 3.2)	—
Health status			<.001
Excellent, very good	57.3 (55.4 to 59.3)	22.9 (21.1 to 24.6)	—
Good	31.8 (30.1 to 33.5)	33.6 (31.7 to 35.4)	—
Fair, poor	10.9 (9.8 to 11.9)	43.5 (41.3 to 45.8)	—
High blood pressure	48.4 (46.4 to 50.5)	70.2 (68.1 to 72.4)	<.001
High cholesterol	48.2 (46.0 to 50.3)	62.2 (59.9 to 64.6)	<.001
Arthritis	41.5 (39.4 to 43.7)	70.0 (67.8 to 72.2)	<.001
Heart disease	23.0 (21.3 to 24.8)	43.9 (41.3 to 46.4)	<.001
Diabetes	12.9 (11.6 to 14.3)	27.6 (25.4 to 29.7)	<.001
Region			.221
Northeast	18.6 (16.4 to 20.9)	17.4 (15.0 to 19.7)	—
Midwest	22.3 (20.2 to 24.4)	22.4 (20.2 to 24.6)	—
South	36.3 (33.5 to 39.2)	39.1 (36.1 to 42.1)	—
West	22.8 (20.8 to 24.7)	21.1 (18.3 to 24.0)	—
Year			.008
2015	19.7 (18.1 to 21.3)	21.4 (19.4 to 23.4)	—
2016	19.2 (17.9 to 20.5)	22.0 (20.3 to 23.8)	—
2017	19.3 (18.1 to 20.5)	18.9 (17.6 to 20.3)	—
2018	20.8 (19.3 to 22.3)	18.9 (17.3 to 20.4)	—
2019	21.1 (19.6 to 22.5)	18.8 (17.1 to 20.5)	—

a Weighted total N = 21 046 285. Percentages and 95% CIs were adjusted for survey design (probability weight, primary sampling unit, strata). CI = confidence interval.

**Figure 1.**
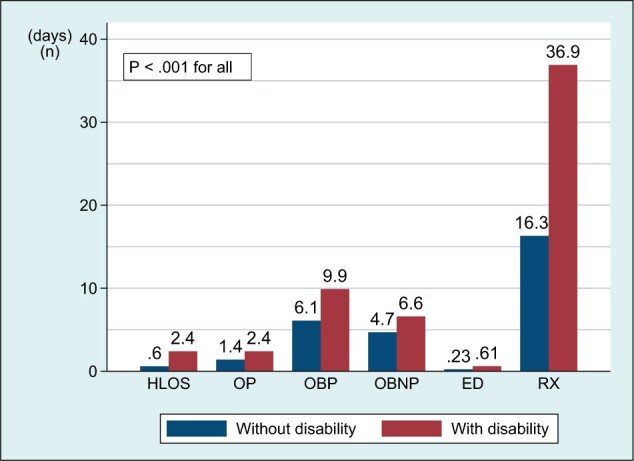
Health services utilization for 6 service categories among cancer survivors (n = 9359). Days and numbers were adjusted for survey design (probability weight, primary sampling unit, strata). ED = emergency department; HLOS = hospital length of stay; OBNP = office-based nonphysician; OBP = office-based physician; OP = outpatient; RX = prescription.

**Figure 2.**
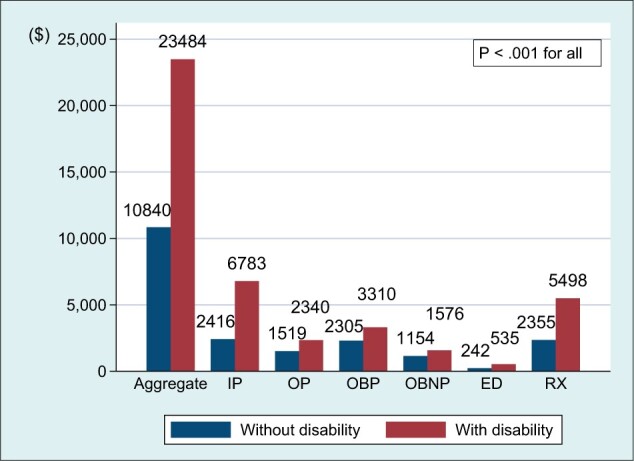
Total medical costs for aggregate services and 6 service categories among cancer survivors (n = 9359). Aggregate services included these 6 services plus home health care, dental care, and other medical equipment and services. Dollars were adjusted for survey design (probability weight, primary sampling unit, strata). ED = emergency department; IP = inpatient; OBNP = office-based nonphysician; OBP = office-based physician; OP = outpatient; RX = prescription.

**Figure 3.**
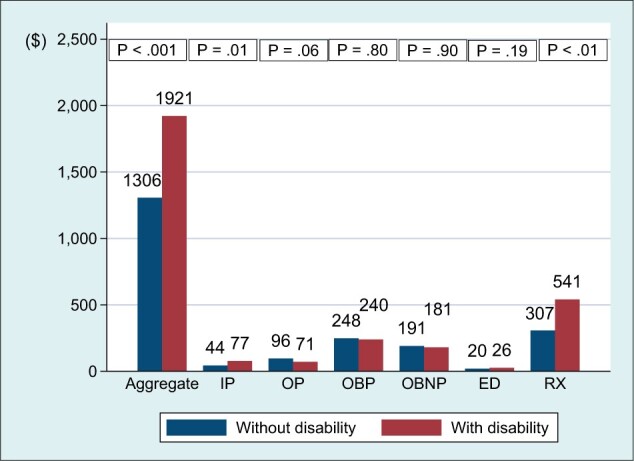
Out-of-pocket medical costs for aggregate services and 6 service categories among cancer survivors (n = 9359). Aggregate services included these 6 services plus home health care, dental care, and other medical equipment and services. Dollars were adjusted for survey design (probability weight, primary sampling unit, strata). ED = emergency department; IP = inpatient; OBNP = office-based nonphysician; OBP = office-based physician; OP = outpatient; RX = prescription.

The presence of a disability among cancer survivors was associated with greater health services utilization for each service category (Figure 1) and total medical costs of aggregate services and costs by service category (Figure 2) than for those with no disability. Compared with those without a disability, those with a disability had greater out-of-pocket costs for aggregate services, inpatient care, and prescription drugs (Figure 3) (*P *<* *.01 for all).

### Adjusted multivariable regression analyses

#### Health services utilization

Table 3 shows the association of having a functional disability with health services utilization for a categorized total number of disabilities (model 1) and 6 binary variables for each disability (model 2), adjusting for all other explanatory variables.

**Table 3.** Association of functional disability with health services utilization among cancer survivors (n = 9359)^a^^,^^b^

	Hospital LOS, average marginal effect (95% CI), d	Outpatient department, average marginal effect (95% CI), No.	Office-based physician, average marginal effect (95% CI), No.	Office-based nonphysician, average marginal effect (95% CI), No.	ED, average marginal effect (95% CI), No.	Prescription, average marginal effect (95% CI), No.
Model 1: total No. of disabilities						
0	0.00 (Referent)	0.00 (Referent)	0.00 (Referent)	0.00 (Referent)	0.00 (Referent)	0.00 (Referent)
1	0.23 (‒0.03 to 0.49)	0.34 (0.01 to 0.67)	1.01 (0.53 to 1.49)	0.51 (‒0.10 to 1.13)	0.09 (0.03 to 0.14)	5.52 (4.18 to 6.86)
2	0.65 (0.27 to 1.02)	0.61 (0.10 to 1.12)	1.87 (1.18 to 2.56)	2.25 (1.02 to 3.49)	0.17 (0.09 to 0.25)	9.32 (7.33 to 11.3)
3	0.94 (0.37 to 1.50)	0.17 (‒0.30 to 0.64)	1.47 (0.63 to 2.32)	1.42 (0.06 to 2.78)	0.19 (0.10 to 0.28)	9.66 (7.41 to 11.9)
≥4	1.14 (0.55 to 1.73)	0.09 (‒0.44 to 0.62)	1.43 (0.51 to 2.35)	0.54 (‒0.72 to 1.80)	0.32 (0.20 to 0.44)	12.1 (9.27 to 15.0)
Model 2: 6 disabilities by type						
Hearing	‒0.12 (‒0.42 to 0.18)	0.17 (‒0.18 to 0.52)	0.23 (‒0.27 to 0.73)	0.22 (‒0.46 to 0.89)	0.03 (‒0.03 to 0.09)	1.66 (0.23 to 3.10)
Vision	0.07 (‒0.28 to 0.42)	‒0.22 (‒0.66 to 0.22)	0.19 (‒0.51 to 0.89)	‒0.61 (‒1.53 to 0.32)	0.01 (‒0.06 to 0.09)	‒1.03 (‒2.88 to 0.82)
Cognition	‒0.31 (‒0.63 to ‒0.01)	‒0.18 (‒0.55 to 0.18)	0.21 (‒0.35 to 0.76)	0.28 (‒0.61 to 1.17)	0.04 (‒0.02 to 0.10)	5.16 (3.42 to 6.90)
Ambulation	0.38 (0.11 to 0.66)	0.07 (‒0.27 to 0.40)	1.03 (0.53 to 1.53)	0.68 (‒0.03 to 1.40)	0.08 (0.03 to 0.13)	5.40 (4.00 to 6.80)
Self-care	0.85 (0.44 to 1.27)	0.00 (‒0.54 to 0.54)	‒0.13 (‒0.95 to 0.69)	‒1.15 (‒2.22 to ‒0.07)	0.08 (0.00 to 0.16)	‒0.46 (‒2.56 to 1.65)
Independent living	0.33 (0.01 to 0.66)	0.36 (‒0.06 to 0.78)	0.58 (‒0.08 to 1.24)	1.61 (0.65 to 2.56)	0.08 (0.02 to 0.15)	3.75 (2.01 to 5.48)

a In multivariable regression analyses, all other explanatory variables listed in Table 2 (age category, sex, race/ethnicity, marital status, education level, employment status, family income, insurance status, general health status, 5 chronic conditions, region, category of calendar year) were adjusted in models 1 and 2.

b Average marginal effects and 95% CIs were adjusted for survey design (probability weight, primary sampling unit, strata). CI = confidence interval; ED = emergency department; LOS = length of stay.

Compared with cancer survivors without a disability, those with 4 or more functional disabilities experienced 1.14 days longer hospital LOS (average marginal effect = 1.14, 95% CI = 0.55 to 1.73), 1.43 more office-based physician visits (average marginal effect = 1.43, 95% CI = 0.51 to 2.35), and 12.1 more prescriptions (average marginal effect = 12.1, 95% CI = 9.27 to 15.0) annually.

By type of disability, compared with those without such disability, disability in self-care was most strongly associated with hospital LOS (average marginal effect = 0.85, 95% CI = 0.44 to 1.27), whereas ambulatory disability was most strongly associated with office-based physician visits (average marginal effect = 1.03, 95% CI = 0.53 to 1.53) and prescription drugs (average marginal effect = 5.40, 95% CI = 4.00 to 6.80). Disability in independent living had the largest impact on the number of office-based nonphysician visits (average marginal effect = 1.61, 95% CI = 0.65 to 2.56). Notably, disability in cognition and self-care substantially decreased inpatient care (average marginal effect = ‒0.31, 95% CI = ‒0.63 to ‒0.01) and the number of office-based nonphysician visits (average marginal effect = ‒1.15, 95% CI = ‒2.22 to ‒0.07), respectively.

#### Total medical costs

Compared with cancer survivors without a disability, the annual total cost of aggregate services was greater by $9537 (average marginal effect = $9537, 95% CI = $5713 to $13 361) for those with 4 or more disabilities (Table 4). Total costs did not always increase monotonically to the number of disabilities. For example, the impact of disability was greatest among those with 4 or more disabilities for inpatient care, whereas the presence of 2 disabilities most strongly affected prescription drug use.

**Table 4.** Association of functional disability with total medical costs among cancer survivors (n = 9359)^a^^,^^b^

	Aggregate services, average marginal effect (95% CI), $	Inpatient care, average marginal effect (95% CI), $	Outpatient department, average marginal effect (95% CI), $	Office-based physician, average marginal effect (95% CI), $	Office-based nonphysician, average marginal effect (95% CI), $	ED, average marginal effect (95% CI), $	Prescription, average marginal effect (95% CI), $
Model 1: total No. of disabilities							
0	0 (Referent)	0 (Referent)	0 (Referent)	0 (Referent)	0 (Referent)	0 (Referent)	0 (Referent)
1	3409 (1876 to 4942)	1069 (205 to 1933)	499 (‒12 to 1010)	173 (‒199 to 545)	139 (‒70 to 347)	107 (32 to 181)	1438 (663 to 2213)
2	9571 (6435 to 12 708)	2405 (1005 to 3806)	1049 (283 to 1815)	930 (341 to 1519)	522 (165 to 878)	232 (104 to 361)	2647 (1485 to 3810)
3	8687 (5521 to 11 853)	2317 (711 to 3924)	421 (‒245 to 1087)	1045 (131 to 1959)	165 (‒203 to 532)	217 (84 to 351)	1897 (895 to 2900)
≥4	9537 (5713 to 13 361)	2988 (1312 to 4663)	‒83 (‒696 to 530)	‒114 (‒663 to 434)	178 (‒363 to 719)	266 (121 to 412)	1312 (372 to 2252)
Model 2: 6 disabilities by type							
Hearing	272 (‒1515 to 2058)	‒340 (‒1251 to 570)	205 (‒274 to 683)	92 (‒338 to 522)	176 (‒38 to 390)	17 (‒58 to 92)	‒7 (‒730 to 717)
Vision	1164 (‒1134 to 3463)	121 (‒989 to 1232)	‒746 (‒1294 to ‒197)	116 (‒474 to 707)	90 (‒268 to 448)	2 (‒89 to 92)	753 (‒335 to 1842)
Cognition	794 (‒1318 to 2906)	‒635 (‒1623 to 352)	423 (‒134 to 980)	‒126 (‒535 to 283)	‒75 (‒352 to 203)	61 (‒14 to 137)	943 (152 to 1734)
Ambulation	3702 (2029 to 5375)	1210 (359 to 2062)	‒2 (‒398 to 394)	156 (‒182 to 495)	99 (‒103 to 301)	94 (21 to 168)	1383 (646 to 2120)
Self-care	3161 (201 to 6122)	1886 (683 to 3088)	38 (‒627 to 702)	‒207 (‒878 to 465)	‒619 (‒937 to ‒300)	45 (‒51 to 142)	‒472 (‒1493 to 548)
Independent living	5046 (2638 to 7455)	1003 (8 to 1998)	335 (‒149 to 820)	693 (183 to 1202)	516 (214 to 819)	76 (‒15 to 166)	411 (‒455 to 1276)

a Aggregate services included the 6 services stated above plus home health care, dental care, and other medical equipment and services.

b In multivariable regression analyses, all other explanatory variables listed in Table 2 (age category, sex, race/ethnicity, marital status, education level, employment status, family income, insurance status, general health status, 5 chronic conditions, region, category of calendar year) were adjusted in models 1 and 2. Average marginal effects and 95% CIs were adjusted for survey design (probability weight, primary sampling unit, strata). CI = confidence interval; ED = emergency department.

By disability type, disability in independent living was most strongly associated with greater total costs of aggregate services (average marginal effect = $5046, 95% CI = $2638 to $7455), followed by disability in ambulation (average marginal effect = $3702, 95% CI = $2029 to $5375) and self-care (average marginal effect = $3161, 95% CI = $201 to $6122). Disability in self-care was strongly associated with greater total costs of inpatient care, whereas ambulation disability was most strongly associated with prescription drugs. Notably, vision and self-care disabilities were associated with lower total costs for visits to an outpatient department and office-based nonphysician, respectively.

#### Out-of-pocket medical costs

Compared with cancer survivors without a disability, having any disability was associated with greater out-of-pocket costs of aggregate services (eg, the average marginal effect of ≥4 disabilities was $639, 95% CI = $79 to $1199), whereas those with 1 or 2 disability conditions had statistically significantly greater out-of-pocket costs for prescription drugs (Table 5). By disability type, disability in independent living and ambulation were strongly associated with greater out-of-pocket costs for aggregate services.

**Table 5.** Association of functional disability with out-of-pocket medical costs among cancer survivors (n = 9359)^a^^,^^b^

	Aggregate services, average marginal effect (95% CI), $	Inpatient care, average marginal effect (95% CI), $	Outpatient department, average marginal effect (95% CI), $	Office-based physician, average marginal effect (95% CI), $	Office-based nonphysician, average marginal effect (95% CI), $	ED, average marginal effect (95% CI), $	Prescription, average marginal effect (95% CI), $
Model 1: total No. of disabilities							
0	0 (Referent)	0 (Referent)	0 (Referent)	0 (Referent)	0 (Referent)	0 (Referent)	0 (Referent)
1	312 (137 to 487)	12 (‒13 to 37)	11 (‒14 to 35)	53 (1 to 105)	22 (‒27 to 71)	11 (1 to 21)	78 (7 to 150)
2	721 (212 to 1231)	36 (‒6 to 79)	‒10 (‒38 to 18)	42 (‒28 to 111)	‒14 (‒66 to 39)	13 (‒2 to 28)	133 (30 to 236)
3	578 (183 to 973)	10 (‒26 to 46)	‒14 (‒45 to 17)	36 (‒57 to 129)	83 (‒58 to 223)	20 (‒2 to 43)	98 (‒2 to 199)
≥4	639 (79 to 1199)	10 (‒30 to 50)	‒38 (‒72 to ‒3)	‒62 (‒126 to 2)	‒7 (‒130 to 116)	4 (‒8 to 17)	13 (‒59 to 84)
Model 2: 6 disabilities by type							
Hearing	182 (‒17 to 381)	‒19 (‒43 to 5)	‒1 (‒27 to 24)	56 (0 to 113)	67 (7 to 126)	‒3 (‒12 to 6)	‒39 (‒100 to 23)
Vision	240 (‒100 to 579)	2 (‒27 to 31)	‒9 (‒54 to 36)	‒51 (‒114 to 12)	3 (‒62 to 69)	9 (‒3 to 21)	58 (‒82 to 199)
Cognition	‒125 (‒368 to 118)	‒16 (‒44 to 12)	‒23 (‒50 to 4)	‒17 (‒73 to 39)	‒6 (‒68 to 56)	2 (‒7 to 11)	46 (‒22 to 114)
Ambulation	239 (7 to 471)	12 (‒10 to 34)	‒2 (‒25 to 22)	20 (‒27 to 67)	‒27 (‒70 to 16)	2 (‒6 to 9)	75 (12 to 137)
Self-care	‒86 (‒500 to 328)	15 (‒21 to 52)	‒57 (‒95 to ‒19)	‒89 (‒167 to ‒11)	‒61 (‒146 to 23)	11 (‒2 to 25)	‒61 (‒148 to 26)
Independent living	525 (123 to 927)	10 (‒16 to 35)	25 (‒8 to 57)	14 (‒61 to 90)	33 (‒33 to 98)	‒2 (‒12 to 9)	13 (‒69 to 95)

a Aggregate services included the 6 services stated above plus home health care, dental care, and other medical equipment and services.

b In multivariable regression analyses, all other explanatory variables listed in Table 2 (age category, sex, race/ethnicity, marital status, education level, employment status, family income, insurance status, general health status, 5 chronic conditions, region, category of calendar year) were adjusted in models 1 and 2. Average marginal effects and 95% CIs were adjusted for survey design (probability weight, primary sampling unit, strata). CI = confidence interval; ED = emergency department.

### Sensitivity analysis

The sensitivity analysis showed that estimates of disabilities were similar in both magnitude and statistical significance when we recategorized age.

## Discussion

This study provides empirical evidence of the impacts on health-care use and medical costs of having a disability among cancer survivors. We found that the prevalence of functional disability was higher among those with than without a cancer history, which motivated subsequent investigation of the impact of having a disability on the health-care experience of cancer survivors. Compared with cancer survivors with no disability, those with disability were more likely to have greater health services utilization and total medical costs for most service types.

The number of cancer survivors in the United States is projected to reach more than 26 million by 2040 (34), making it imperative to understand their ability to maintain normal daily function and financial stability. The importance of this knowledge is highlighted by the recent Consensus Study Report from the National Academies of Sciences, Engineering, and Medicine documenting that cancer-related impairments can lead to functional limitations, which rarely occur as a single problem and often require multicomponent interventions (35). Indeed, 19.6% of cancer survivors had 2 or more disability conditions in our study population, and total and out-of-pocket costs for aggregate services were dramatically greater among those with 2 or more disability conditions than 1 disability. Therefore, understanding the prevalence of functional disability, overall and by type, among cancer survivors and its impact is critical for survivorship care planning and policy interventions to mitigate financial hardship.

Our study makes several novel contributions to the literature. First, although previous studies used a variety of definitions for disability (eg, activities of daily living (36,37), Social Security Disability Insurance (13,38)), we provided estimates of the prevalence of functional disability, collectively and by type, based on the standardized HHS definition (26). Second, prior research largely focused on the association of disability with use of a specific health service (eg, ED) (10,11,13,36,37). We examined the association with various health service categories to provide a more comprehensive perspective of how cancer survivors with functional disability interact with the health-care system and whether this relationship differs by disability. Third, our study provides the first empirical evidence on the potential financial burden associated with disability for cancer survivors, from the perspective of both the health-care system (total costs) and the patient (out-of-pocket costs).

Of note, the relationship between disability and health-care use and costs is complex. Health services utilization and medical costs did not always increase monotonically to the total number of disabilities. Sensory impairment and cognitive decline had no statistically significant impact on health-care use and costs in most health-care categories. Furthermore, for some specific disability conditions, such as cognition and self-care, there could be a negative association with health-care use or costs.

We do not have adequate information to empirically assess the underlying mechanisms. However, 1 potential explanation is that although disability causes poor health status, which in turn increases health-care needs and medical costs (4-6,39), disability itself could be an obstacle to timely and equitable access to care (40,41). The impact of poor health status on health services utilization and costs was greater than that of limited access to care with certain disability conditions, but the overall impact of these 2 opposing forces may be offset among patients with sensory impairment or cognitive decline and result in statistically insignificant results. Alternatively, certain disability conditions could result in replacing 1 type of health service with another. For example, cancer survivors who have a self-care-related disability used fewer office-based nonphysician visits, which may imply that they replaced those visits with home health care. Further investigation is necessary to understand differential mechanisms by type and severity of disability and its impact on health-care use, medical costs, and long-term health outcomes.

Cancer survivors often have other chronic conditions (28,42,43) that could lead to disability (44,45). One report found that the presence of chronic conditions substantially increased medical costs among cancer survivors (28), but specific types of disability were not included in the analysis. Our study showed a greater proportion of cancer survivors with disability had any of 5 chronic conditions prevalent among cancer survivors (Table 2), but the correlation coefficients between each of 6 disability conditions and each of 5 chronic conditions were low, ranging from .10 to .38 (*P *<* *.001 for all). Despite the statistical significance of the correlations, the presence of disability had an independent impact on health services utilization and medical costs, even when we adjusted for both disability and chronic conditions as well as various sociodemographic factors in our multivariable regression analyses. This finding suggests that survivorship care planning should take into account patients’ disability profiles in addition to their coexisting chronic conditions to fully comprehend the possible health-care need and financial hardship.

Increase in health services utilization and medical costs associated with any functional disability was greater, albeit statistically insignificant, among individuals with than without a cancer history in most service categories (results not shown). For example, the increase in total prescription costs associated with any disability was greater among individuals with a cancer history than those without by $747 (95% CI = ‒$695 to $2189), implying a greater financial burden for some cancer survivors. Many cancer survivors already experience financial hardship driven by high treatment costs, especially when they are uninsured or underinsured (2,3,17,46). The additional increase in medical costs resulting from disability could be profound, considering that approximately 40% of American adults could not cover a modest unexpected medical expense of $400 (47), and cancer survivors with disability in our study were less likely to be employed and more likely to have lower family income.

Some limitations of this study warrant discussion. First, functional disability, health services utilization, and costs were self-reported in MEPS-FC data and, therefore, are subject to reporting or recall bias. Nevertheless, studies have shown agreement between household survey and medical records (48,49). Second, MEPS-FC provides cross-sectional annual data that do not include individuals who died in any given year, potentially yielding an underestimate of the overall prevalence of disability and the impact of disability on outcomes among cancer survivors (50). Third, MEPS-FC does not collect detailed information about the cancer diagnosis, such as date of diagnosis and disease stage, or about the disability, such as age of occurrence and cause of disability. Thus, we could not tell the sequence of events to determine whether disability predated cancer diagnosis or was a result of impairment from cancer treatment. Fourth, although smoking status is a potential confounder, we did not adjust for this status in our analyses because MEPS-FC collected current smoking status only, not former smoking history.

In summary, functional disability intensified health-care needs and aggravated financial burden among cancer survivors, and the impact varied by type and total number of disabilities. These findings suggest that health policy for cancer survivors with functional disability should be designed based on an understanding of the underlying mechanisms of patients’ health-care needs and be tailored by type and severity of disability to improve access to care and mitigate financial hardship in more effective ways in cancer survivorship planning.

## Supplementary Material

pkad059_Supplementary_Data

## Data Availability

MEPS-FC is sponsored by the Agency for Healthcare Research and Quality and is publicly available at https://meps.ahrq.gov/mepsweb/.
