# Supplementary material for: Impact of functional disability on health-care use and medical costs among cancer survivors
Source: JNCI Cancer Spectr. 2023 Aug 16;7(5):pkad059. doi: 10.1093/jncics/pkad059 (PMC10505255; doi:10.1093/jncics/pkad059)

Supplementary Table. U.S. Department of Health and Human Services implementation guidance on data collection standards for disability status

Questionnaire	Yes	No
1. (Hearing) Are you deaf or do you have serious difficulty hearing?		
2. (Vision) Are you blind or do you have serious difficulty seeing, even when wearing glasses?		
3. (Cognition) Because of a physical, mental, or emotional condition, do you have serious difficulty concentrating, remembering, or making decisions? (5 years old or older)		
4. (Ambulation) Do you have serious difficulty walking or climbing stairs? (5 years old or older)		
5. (Self-care) Do you have difficulty dressing or bathing? (5 years old or older)		
6. (Independent living) Because of a physical, mental, or emotional condition, do you have difficulty doing errands alone such as visiting a doctor's office or shopping? (15 years old or older)		

Notes:

(i) The six item set of questions used on American Community Survey and other major surveys to gauge disability is the data standard for survey questions on disability. Note the age thresholds for survey participants for the different disability questions.

(ii) Resource: Implementation Guidance on Data Collections Standards for Race, Ethnicity, Sex, Primary Language, and Disability Status. U.S. Department of Health and Human Services. Office of the Assistant Secretary for Planning and Evaluation. Available at: http://aspe.hhs.gov/datacncl/standards/ACA/4302

Supplementary Figure. Selection of applicable study population for cancer survivors without and with disability from 2015 to 2019 Medical Expenditure Panel Survey Household Component Full Year Consolidated File (MEPS-FC)

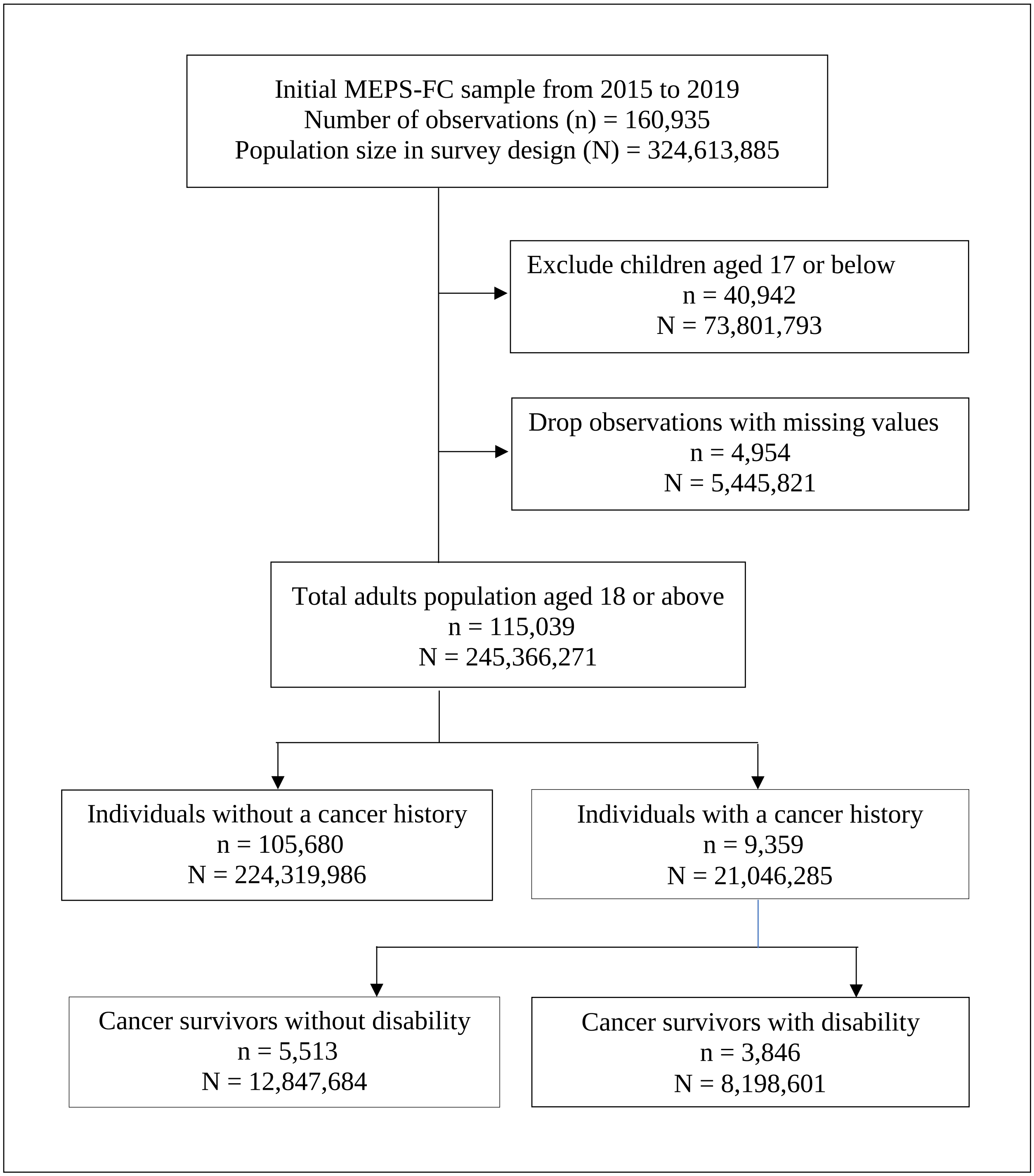

Supplement: pkad059_Supplementary_Data [file pkad059_supplementary_data.pdf]
